# Assessing Environmental Hotspots and Sustainable Development Goal Alignment in Food Production in India

**DOI:** 10.1002/fsn3.70573

**Published:** 2025-07-18

**Authors:** S. U. Parvathy, Vysakh Kani Kolil, Krishnashree Achuthan

**Affiliations:** ^1^ Center for Cybersecurity Systems and Networks Amrita Vishwa Vidyapeetham Kollam Kerala India

**Keywords:** basket of products, environment, food production, India, life cycle assessment, sustainable development goals

## Abstract

The global food system's unsustainable practices present considerable environmental risks while fulfilling essential nutritional needs. This study presents a comprehensive evaluation of the environmental impacts associated with commonly consumed food items in India. It focuses on eighteen representative products, grouped into five major food categories, and uses a production‐focused Life Cycle Assessment (LCA) approach to analyze their environmental performance. Key environmental impacts, such as greenhouse gas emissions, land use, water consumption, and biodiversity loss, are characterized based on per capita consumption. Primary environmental hotspots in the life cycle of Indian household goods, particularly various food product categories, are identified. The findings are contextualized within several Sustainable Development Goals (SDGs), specifically SDG 3, SDG 6, SDG 13, SDG 14, and SDG 15. Scenario analyses present four potential dietary shifts balancing high nutritional value with reduced environmental impact, suggesting that increasing the consumption of millets and sorghum could reduce environmental impacts by 62%–79%. This study underscores the critical role of dietary choices and sustainable agricultural practices, along with policy directives, to achieve sustainable production and consumption while enhancing nutritional adequacy.

## Graphical abstract

This study evaluates the environmental impacts of 18 commonly consumed food items in India using a production‐based LCA, highlighting key hotspots in greenhouse gas emissions, land and water use, and biodiversity loss. It aligns findings with SDGs 3, 6, 13, 14, and 15, and proposes dietary shifts, especially toward millets and sorghum, that could reduce impacts by up to 79%. The results emphasize the importance of sustainable diets and agricultural practices for environmental and nutritional gains.

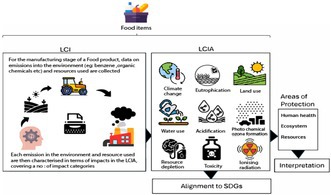

## 1 Introduction

In the face of mounting global environmental challenges, the food production sector stands as a critical intersection between human sustenance and ecological sustainability (Springmann et al. 2018). The global food system is a significant driver of environmental change, accounting for a substantial proportion of global greenhouse gas emissions, land use, freshwater use, and biodiversity loss (Imran et al. 2023; Lazaro et al. 2019). According to recent data, food production alone contributes to more than a quarter (26%) of global greenhouse gas emissions, utilizing half of the world's habitable land for agriculture. Moreover, a substantial portion of global freshwater withdrawals, around 70%, is attributed to agriculture, with agricultural activities responsible for a staggering 78% of global ocean and freshwater eutrophication. Livestock production, in particular, exerts immense pressure on biodiversity, outweighing wild mammals by a factor of 15‐to‐1. This disparity underscores the significant impact of agricultural practices on global ecosystems. Conventional agricultural practices heavily reliant on synthetic inputs such as fertilizers, herbicides, and pesticides exacerbate deforestation, air, soil, and water pollution, and contribute significantly to greenhouse gas emissions (Eskandari et al. 2022). The adverse effects of food production extend beyond greenhouse gas emissions and land use. They encompass a spectrum of environmental concerns such as land degradation, loss of wild and agro‐biodiversity, water depletion, pollution of soil and water, and emergence of pest resistance (Baba and Adamu 2021). These impacts are substantiated by extensive research across various agricultural domains (Balogh and Jámbor 2020). As such, it has become increasingly important to explore sustainable practices or a paradigm shift that can mitigate the adverse effects of food production (Xue et al. 2021).

The present patterns in food production and consumption are being increasingly seen as unsustainable, while yet meeting the fundamental human need for nutrition (He et al. 2018; You et al. 2022). Sustainable Development Goal (SDG) 2 (Zero Hunger), which is to eradicate all kinds of hunger and malnutrition by 2030, is one of the main priorities of most countries (Delabre et al. 2021; Onoja and Adione 2020). India, being the world's most populous country, is an illustrative example of where rapid demographic expansion intensifies pressure on food systems to not only feed a growing population but to do so in an environmentally sustainable manner (Paul et al. 2023). With a diverse array of dietary preferences influenced by regional, religious, and cultural factors, the Indian food system presents a complex tapestry of agricultural practices and consumption patterns (Das 2024; Manik et al. 2024). The challenge lies not only in addressing the environmental impacts of these practices but also in aligning them with the broader objectives encapsulated in the SDGs. The urgency of this challenge is further magnified by the projected trends in population growth and urbanization (Barrett 2021), which are expected to place additional pressures on India's food systems (Mehta et al. 2024). Over two billion people globally, nearly half of whom reside in India, struggle with hidden hunger or micronutrient insufficiency. These deficiencies are brought on by a diet high in cereal and low in variety and nutrients. Thus, to guarantee food and nutritional security for the growing population, sustainable production systems can be a good option. Millets and Sorghum, often referred to as “Nutri‐cereals,” are climate‐resilient crops with an improved nutritional profile and greater performance under marginal growing conditions. As a result, they can contribute to the sustainable achievement of nutritional security (Nagaraj and Amarajothi 2017; Porwal et al. 2023).

Life Cycle Assessment (LCA) methodologies are widely utilized to evaluate the environmental impacts associated with all stages of a product's life, from raw material extraction through production, use, and disposal (Parvathy et al. 2025). Specific environmental hotspots are stages or processes within crop cultivation that contribute disproportionately to the overall environmental impact, thereby highlighting critical areas for reducing environmental harm (Hunfeld et al. 2023). For instance, a study focusing on maize cultivation revealed fertilization as the most influential factor, followed by irrigation (Wang et al. 2023). Another comparative study on tomato cultivation in India underscores the environmental benefits of organic farming, showcasing significant reductions in greenhouse gas emissions and other impact categories compared to conventional methods (Kumar et al. 2024).

Central to evaluating sustainability and guiding decision‐making across various sectors is the basket of products (BoP) approach (Castellani et al. 2021). This approach involves selecting a representative set of food items based on their prevalence in the average Indian diet and analyzing their production phases from raw material extraction to consumer use. By examining multiple products within specific categories or sectors, such as household goods or bakery products, the BoP approach facilitates a holistic understanding of consumption patterns and production processes. Through identifying environmental hotspots and areas for improvement (Barbhuiya and Das 2023), stakeholders can make informed decisions to advance sustainability objectives. This methodology proves instrumental in conducting LCAs, offering a comprehensive perspective on the environmental impact associated with a range of products.

Despite the increasing amount of research on evaluating the environmental impact of individual products, there is still a lack of studies that take a consumption‐focused approach to fully examine the global environmental impact of food production (Nguyen et al. 2022). Thus, creating policies that guarantee food security, environmental sustainability, and socioeconomic fairness requires an awareness of the environmental effects connected to food production.

This article seeks to explore the environmental footprints of food production within Indian households, focusing on how these activities intersect with and impact various ecological dimensions and SDGs. The study makes several key contributions. First, the study systematically evaluates commonly consumed food items in Indian households through BoP methodology using a production‐focused approach and LCA methodology. Second, environmental impacts such as greenhouse gas emissions, land use, water consumption, and biodiversity loss are characterized based on per capita consumption. Third, primary environmental hotspots in the life cycle of Indian household goods, focusing on food product categories, were ascertained. Fourth, the findings are contextualized within several SDGs, and the impact on SDG 3, SDG 6, SDG 13, SDG 14, and SDG 15 is presented. Fifth, four possible scenarios that can address the dual goals of high nutritional value and lower environmental impact are presented. The study outlines how current consumption patterns impact these goals.

## 2 Methodology

The formulation of the BoP approach is a response to the necessity of analyzing and tracking Indian consumption habits and their worldwide impact. This is done with the aim of transitioning toward consumption behaviors that are more efficient in their use of resources and have impact (Sala and Castellani 2019). The BoP indicators precisely measure the environmental effects of Indian consumer behaviors by utilizing manufacturing data and consumption statistics (Castellani, Beylot, and Sala 2019; Castellani, Hidalgo, et al. 2019; Terenteva et al. 2023). The selected BoP is regarding Food. The BoP in relation to human nutrition is of great importance, as the production and consumption of food and beverages account for more than one‐third of the total environmental impact resulting from private consumption (Notarnicola et al. 2017). The Basket allows for comparative analysis to identify best practices, technological innovations, and opportunities for improvement.

The key steps for determining the BoP's environmental effects include (Castellani, Beylot, and Sala 2019; Castellani, Hidalgo, et al. 2019):

- Selecting the representative products, in terms of high consumption
- Calculation of the environmental impacts, adopting the ReCiPe methodology

The geographical area of the assessment is India for the BoP. The study used a consumption‐based methodology. This necessitates the consideration of the environmental repercussions caused, whether directly or indirectly, by the consumption patterns of Indian consumers, including the impacts stemming from the production process of items.

### 2.1 Selection of the Basket of Products

**FIGURE 1:**
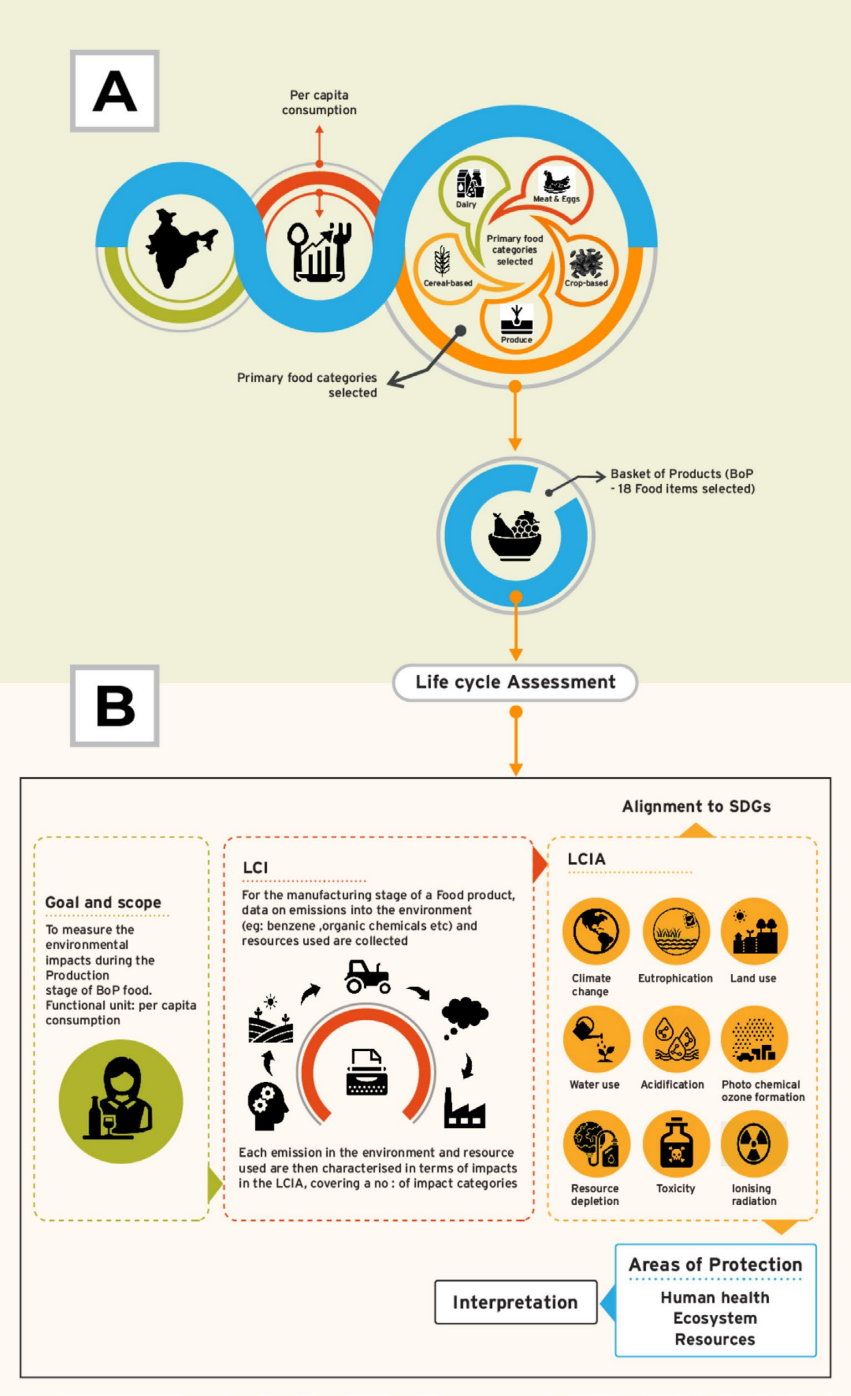
Study design.

Designing a basket of food products or BoP is done based on per capita consumption that helps with understanding the food consumption patterns of specific consumers (Reale et al. 2019). Many studies have used the BoP technique (Baldassarri et al. 2017; Castellani, Hidalgo, et al. 2019). Through the examination of per capita consumption data, it is feasible to determine the food items that are most frequently consumed, reflecting the preferences and behaviors of specific groups (Authority 2009), which in this case is India. The approach adopted to build the basket of products included the following steps (Figure 1A).

1. Defining five primary food categories that included meat and eggs, dairy products, crop‐based products, cereal‐based products, and produce.
2. Products with the highest mass value per capita usage were chosen among these primary food group categories. The data on apparent consumption were acquired from literature sources and specific Food and Agriculture Organization databases.
3. The above process resulted in 18 food products to be included as part of BoP (Table 1).

**TABLE 1:** Selected basket of products and per capita consumption.

Product groups	Basket products	Total average Indian consumption of the food product (in million metric tons)	Per capita consumption of the food product (in kg)
Meat and eggs	Poultry meat	4407.24	0.003
	Beef meat	3000	2.14
	Pork meat	292.5	0.209
	Eggs	180	9.0
Dairy products	Milk	289.486	207.49
	Cheese	280	0.2
	Butter	6.68	4.771
Cereal‐based products	Bread	9.56	6.83
	Wheat	104.0	74.29
	Rice	100.0	21.42
	Millet	13	9.28
	Sorghum	4.018	2.87
Crop‐based products	Sugar	30.51	21.79
	Sunflower oil	1.8	1.286
	Mustard oil	4	2.86
Produce	Potatoes	41.6	25.4
	Onions	22.82	16.3
	Beans	28.56	20.0

There was a noticeable increase in the consumption of these products over the last 10 years. However, to validate the outcomes from an Indian standpoint using a BoP model, the study was conducted.

### 2.2 Life Cycle Assessment

LCA is a rigorous approach employed to assess the ecological consequences of a product, process, or service throughout its complete life cycle (Mattila et al. 2012; Palazzo et al. 2020). A product or service's life cycle includes all of its stages, beginning with the extraction of raw materials and continuing through manufacturing, distribution, use, and, at the end of its useful life, disposal (Kar et al. 2024; Rugani et al. 2019). The application of LCA was employed to evaluate the possible environmental consequences of BoP initiatives in the food sector.

The analysis of environmental impacts utilized the LCA methodology, which establishes a direct connection between mass and energy balances and environmental impact (Morales et al. 2015). This approach evaluates the potential environmental impacts of the proposed schemes from a technological perspective. The approach utilized was ISO 14040, which is a standard set by the International Organization for Standardization in 2006 (ISO 2006). This methodology has several parts (Figure 1).

Definition of scope: Conducting an LCA on a BoP of food products to quantify and compare their environmental impacts across the production stage of the food items (Bruhn et al. 2023; Langkau et al. 2023). A production‐centric approach in LCA of food products is crucial for identifying opportunities to improve sustainability (de Bruyn 2019). It focuses on the manufacturing and production stages of a product or service to identify opportunities for improvement and reduce environmental impacts (Enyoghasi and Badurdeen 2021). In the end, this strategy contributes to the creation of more sustainable food production systems by assisting food corporations in minimizing their negative effects on the environment, streamlining operations, and making wise supply chain decisions (Falcone et al. 2023). This study considered only the manufacturing stage of the products, excluding the transportation and management of waste during the stage.

Purpose: Evaluate and compare the environmental impacts of different food items within the basket, identifying hotspots and areas for improvement (Liu et al. 2023).

Life cycle inventory (LCI) analysis of the study systems: The main data sources used for building the inventories are Ecoinvent v3 (Frischknecht et al. 2007; Weidema et al. 2013) and Agri‐footprint version 5.0 (van Paassen et al. 2019), complemented with data from the literature. Only the manufacturing stage was considered for doing this analysis. The unit of analysis (functional unit) of the approach is the consumption in India in the area of food.

Life cycle impact assessment (LCIA): The selection of impact categories, category indicators, and characterization models for LCIA was based on existing literature pertaining to this specific type of system (Hauschild 2018). Subsequently, LCI findings were allocated to the chosen effect categories using categorization, and the category indicators were then computed through characterization (Rosenbaum et al. 2018). The execution of this step was performed via the commercially available software application SimaPro.

Result interpretation: During this phase, the analysis of the inventory and the assessment of its impact are examined to conclude the objectives and scope. The study found environmental burdens and areas for improvement (Zampori et al. 2016).

### 2.3 Damage Assessment

The damage assessment phase of LCA is a crucial step that converts environmental intervention inventory data (e.g., emissions, resource consumption) into potential effects on ecosystems, human health, and resource availability. In this study, damage assessment is done for the major contributor or food product that produces high value for the impact categories considered. The characterized results from LCIA are further processed to estimate their contributions to damage categories.

### 2.4 Impact on Sustainable Development Goals

Evaluating the environmental impact of goods and services is vital for reaching the SDGs. This study used the framework developed by (Castellani, Beylot, and Sala 2019) to ensure a more complete and robust evaluation of the impacts, addressing SDGs, mainly the ones addressing ecosystem quality and human health. The major contributors of food products to the 18 impact categories considered were identified and compared with alternatives. To understand the effects on the impact category on SDGs for the products considered, a scenario analysis was conducted. The purpose of the scenario analysis is to present multiple potential future development paths that yield different outcomes and associated implications and to evaluate the effects of possible future events on system performance by considering various alternative scenarios. Similarly, we considered four scenarios and conducted the analysis.

### 2.5 SimaPro Software

The LCA is conducted using the SimaPro software, which was specifically designed and built for this purpose by PRé Consultants in 2003. To calculate the environmental indicators (warming potential, acidification, eutrophication, toxicity, etc.) ReCiPe 2016 Endpoint (H) was selected as the impact assessment model for the analysis. ReCiPe is a method for LCA impact assessment that translates emissions and resource extractions into environmental impact scores using characterization factors (Huijbregts et al. 2017). It includes both midpoint (problem‐oriented) and endpoint (damage‐oriented) indicators. The method, along with 18 impact categories, was used because they are the most used in LCA studies due to the uncertainty associated with endpoint impact categories (Chen, Matthews, and Griffin 2021; Feng et al. 2023). The impact categories also align well with SDGs, particularly 3, 6, 13, 14, and 15, due to their relevance in evaluating environmental and health‐related impacts of food systems.

## 3 Results and Discussion

### 3.1 Interpretation of Results

#### 3.1.1 Characterization Results

The characterization results in the LCIA step offer essential insights into the environmental consequences linked to the apparent consumption of different goods within the BoP food category in India in the reference year of 2023. The LCIA process has multiple processes, such as defining the system boundary, feeding data into software, selecting the functional unit, and picking the desired effect model. The process of LCIA entails the compilation, analysis, and interpretation of classified data to assess its environmental effects (Rosenbaum et al. 2018).

**FIGURE 2:**
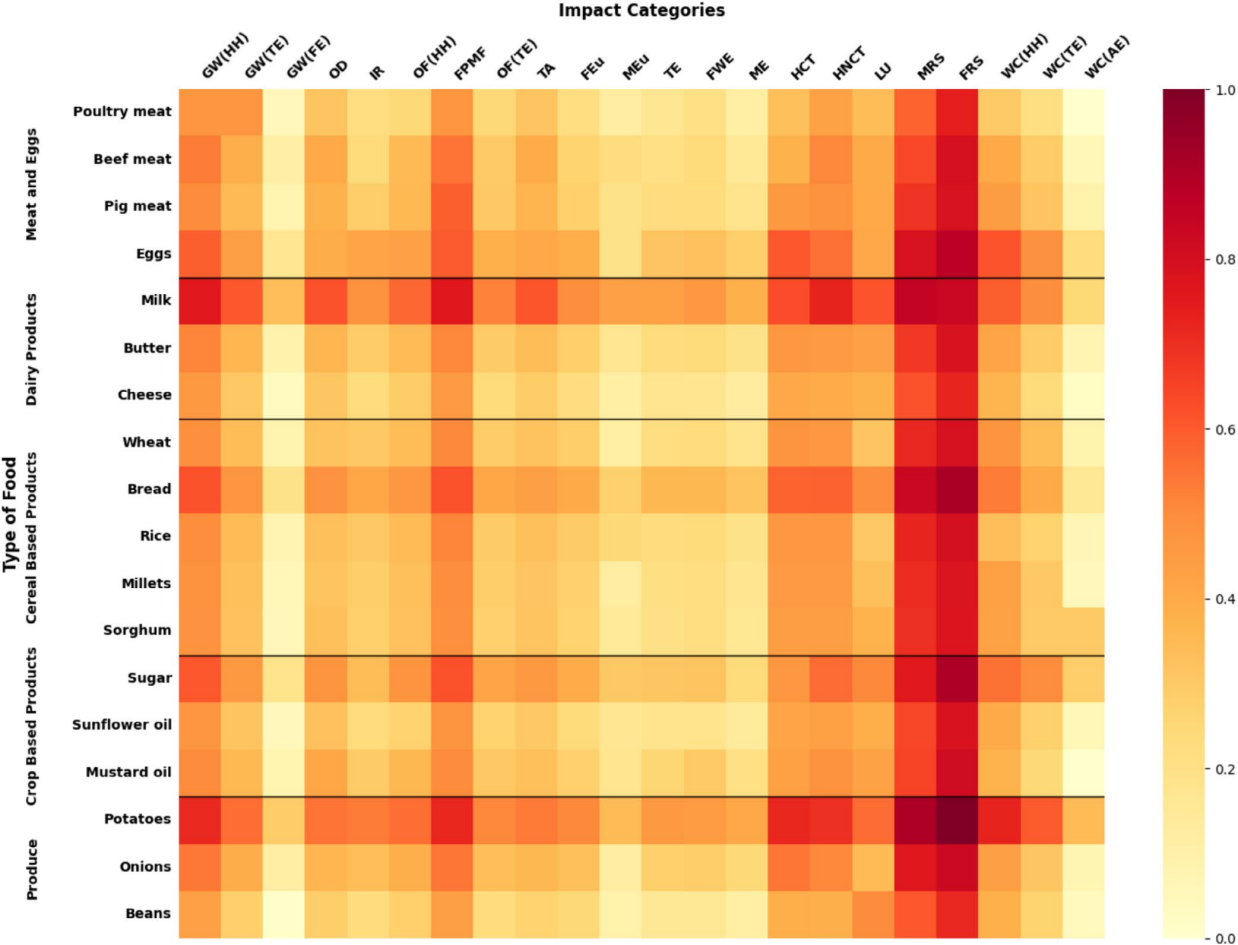
Heat maps of environmental impacts of food products.

The study utilizes various effect categories, as outlined in Table 2, which cover a wide range of environmental issues. Every impact category is quantified using distinct units, such as kilograms of CO_2_ equivalents for GW or kilograms of SO_2_ equivalents for AD. The data shown in Table 3 offer a thorough analysis of the environmental consequences related to the consumption of 18 distinct goods in the BoP food category in India during the year 2023. The results encompass a range of effect categories, such as greenhouse gas emissions, water usage, land use, and others (Figure 2).

**TABLE 2:** Impact categories of the ReCiPe 2016 Endpoint H V1.05 database.

Impact category	Unit	Abbreviation
Global warming	kg CO_2_eq	GW
Ozone depletion	kg CFC‐11 eq	OD
Human toxicity potential	kg 1,4‐dB eq	HTP
Fresh water aquatic ecotoxicity	kg 1,4‐dB eq	FE
Marine aquatic ecotoxicity	kg 1,4‐dB eq	ME
Terrestrial ecotoxicity	kg 1,4‐dB eq	TE
Photochemical oxidation	kg C_2_H_4_ eq	PO
Acidification potential	kg SO_2_ eq	AD
Eutrophication potential	kg PO_4_ eq	ETP
Ionizing radiation	kg U_235_	IR

**TABLE 3:** Impact assessment values of the ReCiPe 2016 Endpoint H V1.05 database.

Impact category	Type of food					
	Wheat	Bread	Beans	Butter	Cheese	Chickpea
GW (human health)	1.31E‐5	0.00186	1.31E‐6	2.87E‐5	3.44E‐6	5.20E‐6
GW (terrestrial ecosystems)	3.95‐E	5.62E‐6	3.95E‐9	8.65E‐8	1.04E‐8	1.57E‐8
GW (freshwater ecosystems)	1.80E‐12	1.53E‐10	1.08E‐13	2.36E‐12	2.83E‐13	4.28E‐13
OD	1.99E‐8	8.17E‐6	5.03E‐9	8.89E‐8	1.12E‐8	1.44E‐8
IR	8.98E‐9	6.37E‐7	5.18E‐10	5.85E‐9	5.36E‐10	3.65E‐10
Ozone formation, human health	3.87E‐8	4.42E‐6	3.52E‐9	4.77E‐8	5.56E‐9	1.05E‐8
Fine particular matter formation	2.30E‐5	0.00174	1.72E‐6	2.78E‐5	3.22E‐6	3.28E‐6
Ozone formation, terrestrial ecosystems	5.62E‐9	6.39E‐7	5.10E‐10	6.90E‐9	8.05E‐10	1.51E‐9
Terrestrial acidification	1.91E‐8	1.62E‐6	2.43E‐9	4.16E‐8	5.13E‐9	5.29E‐9
Freshwater eutrophication	4.76E‐9	3.78E‐7	1.09E‐9	4.00E‐9	4.25E‐10	2.19E‐9
Marine eutrophication	6.30E‐12	2.98E‐9	2.87E‐12	4.87E‐11	6.18E‐12	1.78E‐11
TE	3.81E‐10	6.77E‐8	3.56E‐11	5.91E‐10	7.05E‐11	5.48E‐11
Freshwater ecotoxicity	5.68E‐10	7.75E‐8	3.44E‐11	6.38E‐10	5.86E‐11	1.68E‐10
ME	9.61E‐11	1.53E‐8	6.51E‐12	1.23E‐10	1.12E‐11	1.03E‐11
Human carcinogenic toxicity	7.20E‐6	0.000461	2.56E‐7	4.56E‐6	4.95E‐7	1.29E‐7
Human non‐carcinogenic toxicity	4.15E‐6	0.000531	2.18E‐7	3.32E‐6	3.26E‐7	1.39E‐6
Land use	1.30E‐8	1.63E‐5	2088E‐8	1.26E‐6	1.62E‐7	2.05E‐7
Mineral resource scarcity	0.0741	7.08	0.00113	0.0166	0.00176	0.00533
Fossil resource scarcity	1.34	146	0.0805	1.02	0.115	0.244
Water consumption, human health	6.53E‐6	6.48E‐5	1.83E‐7	8.60E‐7	9.94E‐8	2.10E‐8
Water consumption. terrestrial ecosystem	4.09E‐8	4.09E‐7	2.24E‐9	5.51E‐9	6.39E‐10	1.74E‐9
Water consumption, aq. ecosystem	2.3E‐12	3.64E‐11	4.20E‐13	1.44E‐12	1.78E‐13	5.23E‐13

Impact category	Type of food							
	Eggs	Milk	Mustard oil	Onions	Pig meat	Potatoes	Rice	Sugar
GW (human health)	0.000596	0.404	1.97E‐5	8.97E‐5	1.91E‐5	0.0655	1.62E‐5	0.00114
GW (terrestrial ecosystems)	1.80E‐6	0.00122	5.95E‐8	2.71E‐7	5.75E‐8	0.000198	4,89E‐8	3.43E‐6
GW (freshwater ecosystems)	4.91E‐11	3.32E‐8	1.63E‐12	7.4E‐12	1.57E‐12	5.4E‐9	1.33E‐12	9.38E‐11
OD	3.01E‐7	0.00169	6.09E‐7	8.81E‐8	1.51E‐7	0.000116	2.88E‐8	6.88E‐6
IR	7.88E‐7	9.17E‐6	6.14E‐9	3.62E‐8	5.1E‐9	6.56E‐5	9.4E‐9	3.90E‐8
Ozone formation, human health	1.33E‐6	0.000282	7.17E‐8	2.08E‐7	6.36E‐8	0.000191	4.32E‐8	6.42E‐6
Fine particular matter formation	0.000869	0.512	2.04E‐5	0.00010	64.82E‐5	0.0967	2.97E‐5	0.00168
Ozone formation, terrestrial ecosystems	1.92E‐7	4.05E‐5	1.08E‐8	3.17E‐8	9.18E‐9	2.77E‐5	6.29E‐9	9.19E‐7
Terrestrial AD	4.8E‐7	0.00129	2.53E‐8	7.26E‐8	9.9E‐8	7.94E‐5	2.64E‐8	3.9E‐6
Freshwater eutrophication	2.38E‐7	1.58E‐5	2.99E‐9	2.49E‐8	3.57E‐9	2.11E‐5	6.12E‐9	3.26E‐7
Marine eutrophication	1.24E‐10	1.37E‐6	6.43E‐11	8.22E‐12	1.25E‐10	4.41E‐8	8.95E‐10	8.33E‐9
TE	1.31E‐8	1.42E‐6	1.49E‐9	3.42E‐9	4.36E‐10	3.53E‐6	5.1E‐10	1.14E‐8
FE	2.00E‐8	4.47E‐6	6.94E‐9	4.83E‐9	5.2E‐10	2.53E‐6	7.62E‐10	1.49E‐8
ME	4.11E‐9	1.90E‐7	2.33E‐10	9.64E‐10	9.17E‐11	5.49E‐7	1.23E‐10	6.85E‐10
Human carcinogenic toxicity	0.00113	0.00277	1.57E‐6	0.00010	3.46E‐6	0.094	4.08E‐06	4.67E‐6
Human non‐carcinogenic toxicity	0.00016	0.131	8.66E‐6	2.68E‐5	9.23E‐6	0.0371	4.49E‐06	0.00023
Land use	5.29E‐7	0.0016	1.15E‐6	4.62E‐8	4.22E‐7	0.000227	9.55E‐9	2.39E‐5
Mineral resource scarcity	1.2	16.1	0.00586	0.481	0.0291	115	0.112	0.422
Fossil resource scarcity	34.7	7.83	3.48	5.48	1.25	4.54E3	1.8	112
Water consumption, Human health	0.00165	0.000618	1.4E‐7	1.86E‐6	2.37E‐6	0.128	3.48E‐8	0.00013
Water consumption, terrestrial ecosystem	1.03E‐5	1.43E‐5	8.95E‐10	1.18E‐8	1.4E‐8	0.000802	2.53E‐9	1.56E‐5
Water consumption, aq. ecosystem	5.54E‐10	1.16E‐9	7.6E‐14	1.1E‐12	2.83E‐12	4.32E‐8	8.19E‐13	4.75E‐9

Impact category	Type of food				
	Sunflower oil	Poultry meat	Beef meat	Millets	Sorghum
GW (human health)	5.48E‐6	5.68E‐6	6.81E‐5	8.81E‐6	7.59E‐6
GW (terrestrial ecosystems)	1.65E‐8	5.68E‐6	2.05E‐7	2.66E‐8	2.29E‐8
GW (freshwater ecosystems)	4.51E‐13	4.68E‐13	5.6E‐12	7.26E‐13	6.26E‐13
OD	2.31E‐8	1.33E‐8	4.66E‐7	1.66E‐8	2.48E‐8
IR	5.51E‐10	3.65E‐10	7.97E‐10	4.82E‐9	3.46E‐9
Ozone formation, human health	2.57E‐9	1.07E‐9	5.34E‐8	3.07E‐8	2.08E‐8
Fine particular matter formation	6.75E‐6	5.39E‐6	0.000119	1.59E‐5	1.26E‐5
Ozone formation, terrestrial ecosystems	2.57E‐9	1.07E‐9	7.67E‐9	4.45E‐9	3.01E‐9
Terrestrial AD	8.23E‐9	1.28E‐8	3.11E‐7	1.44E‐8	1.51E‐8
Freshwater eutrophication	7.84E‐10	3.51E‐10	2.22E‐9	2.87E‐9	2.64E‐9
Marine eutrophication	4.92E‐11	1.04E‐11	4.49E‐10	9.44E‐12	2.22E‐11
TE	7.91E‐11	4.94E‐11	2.4E‐10	2.55E‐10	1.83E‐10
FE	9.79E‐11	1.88E‐10	7.85E‐10	3.72E‐10	3.05E‐10
ME	1.67E‐11	6.07E‐12	2.81E‐11	6.23E‐11	4.35E‐11
Human carcinogenic toxicity	7.83E‐7	3.01E‐8	1.43E‐7	3.12E‐6	1.95E‐6
Human non‐carcinogenic toxicity	1.48E‐6	1.13E‐6	2.7E‐5	2.99E‐6	2.06E‐6
Mineral resource scarcity	2.59E‐7	4E‐8	3.9E‐7	2.72E‐8	1.35E‐7
Land use	0.00431	0.000431	0.00412	0.0496	0.0358
Fossil resource scarcity	0.959	0.194	1.6	0.943	0.725
Water consumption, human health	3.94E‐7	7.39E‐9	4.4E‐7	1.43E‐6	1.06E‐6
Water consumption, terrestrial ecosystem	3.18E‐9	2.5E‐10	5.6E‐9	8.97E‐9	7.76E‐9
Water consumption, aq. ecosystem	7.85E‐13	7.12E‐14	7.63E‐13	5.21E‐13	7.34E‐9

Out of all the products that were examined, dairy items, specifically milk, and tubers such as potatoes, have the highest environmental loads in several impact categories. This discovery suggests that the production and consumption of these foods have substantial resource utilization and environmental consequences. The significant ecological impact linked to dairy and tuber production highlights the need to tackle sustainability issues in these industries to reduce their environmental imprint and encourage the use of more eco‐friendly methods.

Crop‐based products such as mustard oil and sunflower oil exhibit high water consumption values (7.6E‐14 and 7.85E‐13, respectively), primarily due to the significant irrigation required for their optimal production. This is particularly concerning in regions where water scarcity is or might become a critical issue, as highlighted by Adeleke and Babalola (2020). In the context of meat products, the production of pig meat significantly influences the impact category related to ozone formation in terrestrial ecosystems, with values recorded at 9.18E‐9. This impact is largely attributed to the emission of air pollutants such as NO_2_and CO_2_ during the production stage, as confirmed by (Ghinea and Leahu 2018).

Beef meat production also stands out with high impact values in the IR category (7.97E‐10). This high negative value is linked to the extensive treatments used to enhance meat tenderness, which not only harm ecosystems but also pose significant threats to human health, according to Arapcheska et al. (2020). Conversely, the production of poultry meat is marked by high impact values in the water consumption category (97.12E‐14), as noted by Wiedemann et al. (2017).

For cereal‐based products, millets and sorghum show similar results across all impact categories due to the similarity in their production techniques, as observed in many studies (Sunil et al. 2024). However, rice production is associated with high values in the land use and water consumption categories, as reported by Chen et al. (2020) and Feng et al. (2023). Wheat production, on the other hand, reports high values for ME (9.61E‐11), primarily due to the significant presence of nitrogen and phosphorus in the fertilizers used (Pourmehdi and Kheiralipour 2023).

These results highlight the various environmental effects linked to various agricultural crops. The necessity for effective water use methods is highlighted by the high water consumption of oilseed crops and poultry meat. Better emission control methods are required due to the air pollution caused by the manufacturing of pig meat. Sustainable methods of producing meat are essential given the harm that cattle raising does to ecosystems and public health. Comparably, rice's high values for water and land use, combined with wheat's link to ME, highlight the significance of sustainable farming methods and fertilizer management in reducing environmental harm.

The environmental impact associated with foods such as dairy products (e.g., milk) and produce (e.g., potatoes) arises from two main factors. Firstly, these foods have a significant impact since they need a large amount of resources and involve sophisticated production methods. For example, dairy production entails operations such as producing dairy cows, which require significant quantities of water, feed, and land. Similarly, crops such as potatoes necessitate substantial amounts of water and energy during their growth, processing, and distribution, which consequently increases their impact on the environment.

Furthermore, the national consumption rates of these items have a significant impact on their overall environmental footprint. Although a food item may have a relatively smaller environmental burden per unit, its widespread consumption can still have a considerable cumulative impact (Han et al. 2018; Ridoutt et al. 2017; Takacs and Borrion 2020). Consider potatoes; although they may have a lesser environmental impact per unit, their use on a large scale worldwide adds significantly to the overall environmental footprint. On the other hand, the situation of milk consumption in nations such as India, where the annual consumption reaches millions of metric tons, is notable. Although milk has a higher environmental burden per unit, its widespread consumption leads to a significant overall environmental impact.

Tackling the environmental issues related to dairy products and tubers requires the implementation of complex and diverse techniques (Xie et al. 2024). Possible strategies to reduce the environmental effects of dairy farming involve advocating for sustainable methods like pasture‐based grazing (Edwards et al. 2024; Macdonald and Roche 2024; Wall et al. 2024), decreasing dependence on chemical inputs (Chmelikova et al. 2024), and enhancing resource efficiency across the production process (Avalos et al. 2024; Kallioniemi et al. 2024). Similarly, efforts to improve potato production and processing should prioritize the efficient utilization of water and energy, the reduction of chemical inputs (Sunitha et al. 2024), and the promotion of soil conservation techniques (Hemkemeyer et al. 2024; Marjanović et al. 2024; Naz et al. 2024). Furthermore, implementing strategies focused on encouraging more equitable and environmentally friendly eating habits could contribute to lowering the overall consumption levels of certain food items that need a significant amount of resources, thereby mitigating their impact on the environment (Deshmukh and Gutte 2024; Singh 2024).

Ultimately, the environmental impact linked to the production and consumption of commodities such as milk and potatoes stems from their resource‐intensive production methods and their extensive use on both a national and global scale. To tackle these difficulties, it is necessary to coordinate efforts to promote sustainable farming methods, enhance the efficiency of resource utilization, and encourage individuals to adopt diets that are more ecologically friendly. By embracing comprehensive strategies, we can strive to reduce the ecological consequences of food production and consumption, while simultaneously guaranteeing food security and sustainability for future generations.

This information underscores the intricate relationship between consumption patterns, environmental impacts, and food choices in India. It emphasizes the importance of adopting sustainable practices and making informed decisions regarding food production and consumption to mitigate environmental degradation and promote long‐term ecological sustainability.

Cost–benefit analyses are essential tools in agricultural planning, providing practical frameworks to assess the required resource commitment, potential long‐term impacts, and effective pathways for implementation. They help prioritize innovations that balance economic viability with sustainability and resilience, while identifying critical barriers and support mechanisms for adoption. Evaluating agricultural innovations reveals the importance of such balanced decision‐making. Precision agriculture technologies like variable‐rate fertilization, for example, offer productivity and environmental benefits but are economically challenging for smaller farms (Munz 2024), especially those with outdated machinery. For these farms, prohibitive costs and delayed returns make adoption difficult without supportive policies, infrastructure investment, and viable exit strategies when profitability cannot be assured. In water‐scarce regions, water‐saving irrigation technologies like dry cultivation illustrate how slight yield sacrifices can yield significant gains in water conservation and emission reductions (Chen, Li, et al. 2021). Similarly, climate‐resilient crops such as those with drought and flood tolerance demonstrate the importance of food security under climate stress (Acevedo et al. 2020). While these crops enhance resilience, their success is often limited by access to resources like seeds and technical assistance. Practical, targeted interventions addressing these barriers enable more widespread adoption and, ultimately, greater impact. Pesticidal plants present a cost‐effective, eco‐friendly alternative to synthetic pesticides, offering health and environmental benefits. However, processing and application challenges hinder adoption. Here, participatory cost–benefit analysis (Piñeiro et al. 2020) reveals that investments in training and necessary tools can significantly improve uptake, particularly in resource‐limited settings. Finally, sustainable agricultural practices show that aligning economic incentives with ecological benefits drives adoption more effectively. Policies designed to balance both sustainability goals and financial viability make sustainable practices feasible and appealing, especially in sensitive farming contexts (Mkindi et al. 2021).

The current study sets out to examine the effects of food intake in India by systematically identifying representative goods and constructing an inventory based on typical conditions in the country. However, it is crucial to recognize certain limitations within the study to gain a comprehensive understanding of the findings.

#### 3.1.2 Damage Assessment

**FIGURE 3:**
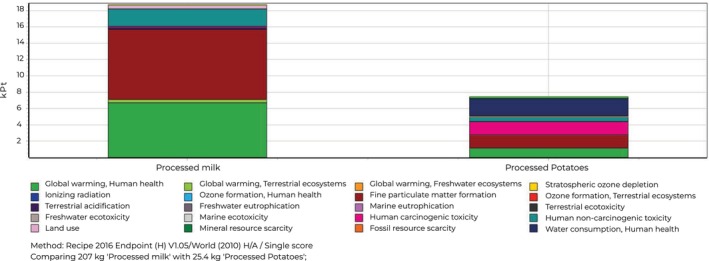
Damage assessment values with impact categories of milk and potatoes.

Figure 3 reveals that milk and potatoes play a substantial role in causing harm in different areas, such as human health, ecosystems, and resource depletion.

Based on the data, milk production has a significant effect on ecosystems, with the Ecosystem harm category accounting for 53% of the total impact. This can be ascribed to multiple sources, including the clearing of forests for grazing land, contamination of water due to the runoff from dairy farms, and the degradation of habitats resulting from intensified agricultural practices. The significant proportion in the Human health category (70.7%) indicates the possibility of negative impacts on human well‐being, potentially caused by substances such as antibiotics or hormones found in milk, allergens in dairy products, or the consumption of unhealthy dairy derivatives (Table 4). Furthermore, the substantial impact of the Resources category (61.2%) highlights the pressure on natural resources that is inherent in milk production. This includes the utilization of water for irrigation and processing, the consumption of energy in transportation and refrigeration, and the degradation of land due to intensive farming methods.

**TABLE 4:** Damage category values of milk and potatoes.

Damage category	Milk	Potatoes
Human health	70.7%	28.4%
Ecosystem	74.2%	24.1%
Resources	61.2%	36.3%

In a similar manner, potatoes have notable effects in different aspects. The significant presence of the resources category (36.3%) highlights the fact that potato production is highly dependent on resources, particularly water for irrigation, chemical inputs such as fertilizers and pesticides, and the deterioration of land due to monoculture farming methods. The Human health category's contribution of 28.4% indicates potential health consequences related to potatoes, such as changes in nutritional value based on farming practices, and the health impacts of processing methods such as frying.

Recent studies highlight how specific farming practices influence the nutritional quality of potatoes, affecting both their macronutrient and micronutrient profiles. For instance, adjusting harvest timing can optimize tuber quality attributes such as starch content, dry matter, and specific gravity (Sharkar et al. 2019). Water management also plays a vital role in potato nutrient density. Crosby and Wang (2021) examined irrigation practices, demonstrating how evapotranspiration can conserve water without sacrificing tuber quality. In terms of nutrient management, Gitari et al. (2018) showed that intercropping potatoes with legumes, such as dolichos, garden pea, and climbing bean, significantly improves nitrogen and phosphorus efficiency. This sustainable practice addresses both soil health and nutrient quality, supporting long‐term agricultural viability. Moreover, research by Eid et al. (2020) explored the combined effect of nitrogen sources and irrigation regimes on potato quality, finding that ammonium nitrate with high irrigation optimizes yield. Biofortification efforts also contribute to improved nutritional outcomes. Zhang et al. (2022) examined the effects of foliar iron fertilizers on potatoes grown in alkaline soils, showing that iron supplementation significantly increases chlorophyll content, photosynthesis efficiency, and iron concentration in tubers. Furthermore, bio‐fertilizers have been shown to boost potato protein levels. A study by Wichrowska and Szczepanek (2020) found that using bio‐fertilizer alongside organic sources, such as farmyard manure, in combination with reduced mineral fertilization increased the protein and essential amino acid content of tubers. Advanced genetic approaches are also being applied to enhance potato nutrition. For example, Tanvir et al. (2022) investigated the use of orphan genes to elevate tuber protein content. This technique allows for protein enrichment without sacrificing yield or altering tuber morphology, a promising development for regions dependent on potatoes as a dietary staple.

Resources are also affected by 36.3% of the total. These studies highlight the diverse effects of milk and potatoes on many environmental and human health factors. It is crucial to take into account these aspects as a whole when making decisions about agriculture, food production, and dietary choices to reduce their negative impacts and foster sustainability in the food system.

### 3.2 Beneficial Contributions of BoP to SDGs

The integration of various food sources such as butter, cheese, and millet aligns with multiple SDGs, supporting health, economic stability, and environmental resilience. Butter offers energy and essential fat‐soluble vitamins (A, D, E, K), aiding undernourished regions (Tripathi 2024) in meeting dietary needs (SDG 2.1, SDG 8.3). Traditional butter production also sustains rural livelihoods by promoting small‐scale dairy farming (SDG 1.4, SDG 8.5; Ulicky et al. 2013). Cheese, similarly nutrient‐dense, provides protein and calcium, vital for dietary diversity and bone health (SDG 2.2, SDG 3.4), while artisanal cheese‐making boosts rural economies (SDG 8.2; Nyamakwere et al. 2021). Millet, a climate‐resilient crop rich in protein and fiber, supports food security and grows in marginal soils, aligning with sustainable agriculture and climate resilience goals (SDG 2.1, SDG 13.1). Staple foods like bread, wheat, and rice are essential to global diets, providing energy and nutrients while supporting local economies. Bread and wheat enhance food security and support small enterprises, particularly in the baking industry (SDG 2.1, SDG 8.3; Noort et al. 2022). Wheat farming, in particular, sustains rural economies by providing stable employment (SDG 8.5). Rice, another staple, is crucial for dietary stability, offering essential calories and nutrients (SDG 2.1, SDG 12.2), and supports livelihoods in developing regions where rice farming is integral to economic resilience (SDG 8.3, SDG 8.5; Yang et al. 2022). Potatoes and onions are affordable, nutrient‐dense crops supporting diverse diets and sustainable agriculture. Potatoes provide carbohydrates, fiber, and vitamins (SDG 2.1, SDG 3.4), and their adaptability to various climates aids small‐scale farmers (SDG 8.3, SDG 12.2). Onions, rich in antioxidants and vitamins, improve dietary quality and are widely cultivated by small farmers, enhancing local food production (SDG 2.1, SDG 3.4, SDG 8.3). High‐protein legumes like beans play a dual role in human nutrition and environmental sustainability (Górska‐Warsewicz et al. 2021). Beans offer essential nutrients and contribute to plant‐based diets (SDG 2.1, SDG 3.4). Their nitrogen‐fixing properties also improve soil health, reducing fertilizer dependence and promoting sustainable farming (SDG 12.4, SDG 15.3). Other agricultural products, such as sugar and cooking oils (mustard and sunflower oil), are economically significant for rural communities, supporting health through diverse dietary options and providing income in developing regions (SDG 8.3, SDG 12.2; Mannucci et al. 2023). Animal products like milk, pork, poultry, and beef contribute to diet diversity, addressing protein and micronutrient needs. Milk supports child growth and nutritional security (Smith et al. 2013), essential for ending malnutrition (SDG 2.2, SDG 3.2), while dairy farming stabilizes rural economies (SDG 1.4, SDG 8.5). Pork, poultry, and beef offer vital nutrients such as protein, B vitamins, and iron, supporting health and addressing malnutrition. These products also contribute to rural employment (Pingali and Plavšić 2022), especially in small‐scale operations, and support sustainable agricultural practices (SDG 8.3, SDG 15.9). Each food source, from crops to animal products, thus addresses specific SDGs by enhancing nutrition, supporting sustainable agricultural practices, and fostering economic growth in rural areas, promoting holistic development across health, economic, and environmental dimensions.

### 3.3 Scenario Analysis and Its Impact on SDGs

**FIGURE 4:**
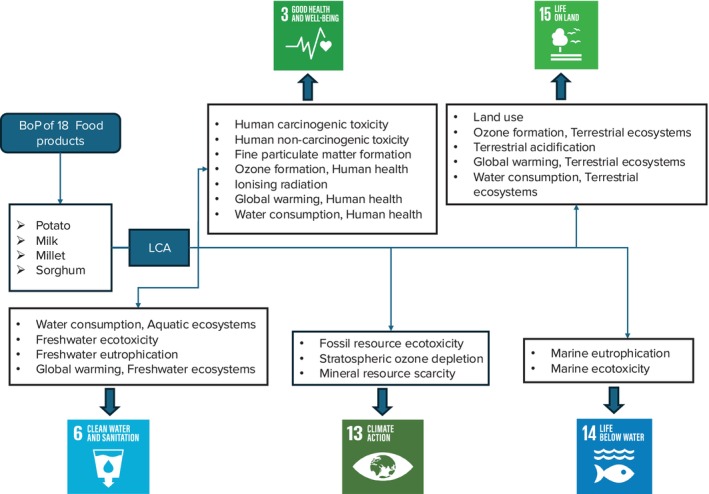
Environmental impact categories and their mapping with SDGs.

Based on the analysis of the most commonly consumed foods, milk and potatoes were found to be the most environmentally detrimental. Previous literature (Hannouf et al. 2023; Backes and Traverso 2022; Wulf et al. 2018) has reported that the impact categories quantitatively influence various SDGs by linking environmental pressures to specific social and ecological outcomes. Frameworks such as ReCiPe 2016 offer a structured way to assess these connections, with studies showing that reductions in impact categories directly contribute to various SDGs. Reducing water consumption directly supports SDG 6, particularly Target 6.4, by preserving freshwater resources, maintaining water availability for essential uses, and reducing stress on ecosystems and treatment infrastructure. Efficient water use ensures long‐term access to clean water, especially in regions facing scarcity. Lowering particulate matter formation supports SDG 3, specifically Target 3.9, by improving air quality and reducing the risk of respiratory and cardiovascular diseases. Cleaner air results in fewer health issues and enhances quality of life, particularly in urban and industrial regions. Reducing greenhouse gas emissions contributes to SDG 13, especially Target 13.2, by mitigating climate change impacts such as extreme weather, sea‐level rise, and ecosystem disruption. Climate action enhances community resilience and protects both human and environmental health. Limiting nutrient runoff and eutrophication aligns with SDG 14, notably Target 14.1, by preventing algal blooms and oxygen depletion in water bodies, which helps protect marine biodiversity and sustain healthy aquatic ecosystems and fisheries. Finally, reducing land degradation, ecotoxicity, and acidification supports SDG 15, including Target 15.3, by protecting soil health, preserving habitats, and maintaining biodiversity. Sustainable land use and pollution control are key to sustaining life on land. While these foods are staples in the Indian diet and cannot be completely replaced, exploring millet and sorghum as complementary additions to traditional diets can enhance nutritional value while meeting broader goals of health and environmental sustainability. Table 5 confirms the nutritional superiority of millets and sorghum over milk and potatoes. We then conducted a scenario analysis (see Table 6) with various intake ratios of millets and sorghum replacing milk and potatoes to quantify their environmental impact and implications for the SDGs. Figure 4 maps the various environmental impact categories to SDGs 3, 4, 6, and others, and the results are discussed below.

**TABLE 5:** Nutritional value per 100 g of potatoes, millets, milk, and sorghum.

Nutrition type	Potatoes	Millets	Milk	Sorghum
Calories	87	300	122	322
Water	77%	—	—	—
Protein	1.9 g	11.2 g	8 g	11 g
Carbohydrates	20.1 g	55 g	12 g	73 g
Sugar	0.9 g	—	12 g	—
Fiber	1.8 g	10.1 g	—	11 g
Fat	0.1 g	4 g	4.6 g	3.3 g
Iron	—	15.2 g	—	4.4 g
Calcium	—	11 g	120 g	28 g

**TABLE 6:** Per capita consumption of products in each scenario.

Products	Scenario 1	Scenario 2	Scenario 3	Scenario 4
Potato (kg)	25.4	19.05	12.7	6.35
Milk (kg)	207.49	155.62	103.75	51.88
Millet (kg)	3.81	4.7625	5.715	6.6675
Sorghum (kg)	2.87	3.5875	4.305	5.0225

The results show that Scenario 1 and Scenario 2 have the highest values across all 18 impact categories, while Scenario 4 has the lowest values. This suggests that Scenario 1 and Scenario 2 have the greatest potential impact on human health, while Scenario 4 has the least potential impact.

#### 3.3.1 Impact on SDG 3 (Good Health and Well‐Being)

**FIGURE 5:**
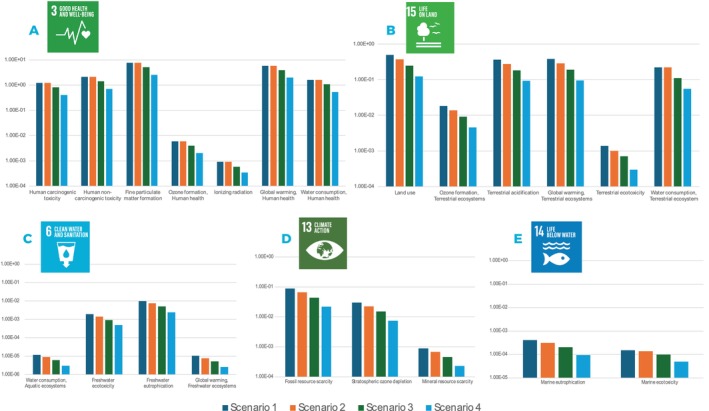
Total environmental impact of potato, milk, millet, and sorghum for each impact category under the SDGs 3, 6, 13, 14, and 15 (Y‐axis refers to the log_10_ of kPt).

In relation to SDG 3, seven out of the 18 impact categories, including human carcinogenic toxicity, human non‐carcinogenic toxicity, fine particulate matter formation, ozone formation (human health), IR, GW (human health), and water consumption (human health), were identified as significant. The study findings indicate that transitioning toward increased consumption of millets and sorghum in Scenarios 3 and 4 can result in substantial reductions across these seven impact categories. Notably, scenario 4 shows remarkable reductions in human carcinogenic toxicity (≈66.65%), human non‐carcinogenic toxicity (≈66.66%), fine particulate matter formation (≈66.66%), ozone formation (human health; ≈66.08%), IR (≈62.40%), GW (human health; ≈66.66%), and water consumption (human health; ≈66.66%; Figure 5A). The high environmental impact values observed in this study can be attributed to specific factors related to potato and milk production.

Potato cultivation involves the use of pesticides and herbicides, which can contaminate the soil and water, potentially leading to exposure and health risks for humans (Kurek et al. 2017; Sookhtanlou et al. 2022). Exposure to pesticides during potato cultivation has been linked to an increased risk of non‐Hodgkin lymphoma in farmers (Jennings et al. 2020). Potato cultivation requires significant water resources, especially for irrigation, which can lead to water scarcity and strain on local water supplies. It can also result in deforestation and habitat destruction, particularly in regions where large‐scale monoculture farming is practiced (Kowalczyk 2019; Singh et al. 2023; Willersinn et al. 2017). Potatoes can absorb heavy metals like cadmium, lead, arsenic, and mercury from contaminated soil or irrigation water, posing health risks, especially for children. Excessive intake of nitrates from potatoes and other foods can lead to adverse effects, particularly in young children (Garrido et al. 2017).

Dairy production involves the use of antibiotics and hormones, which can contaminate the environment and potentially harm human health. Exposure to antibiotics in dairy products increases the risk of antibiotic resistance in humans (Ben et al. 2019). Exposure to pesticides during dairy production can also increase the risk of neurological problems in farmers (Kori et al. 2018). Dairy farming requires substantial water resources for animal feed and irrigation, straining local water supplies. It is also a significant source of greenhouse gas emissions, primarily due to the methane produced by livestock and the energy‐intensive processes involved in milk processing and transportation. Dairy farming can lead to deforestation and habitat destruction as large areas of land are allocated to grazing and feed crop production (Kumar et al. 2023; Rezaei et al. 2018).

Research indicates that potatoes have a shallow root system with lower efficiency in extracting water from the soil compared to other crops, making them particularly vulnerable to water stress and drought conditions (Jacques et al. 2020; Li et al. 2023; Nasir and Toth 2022). Potato cultivation's reliance on irrigation for water supply, especially in regions where water resources are limited, can exacerbate water scarcity issues. The need for substantial water resources for potato irrigation, combined with the crop's susceptibility to drought, can strain local water supplies. Furthermore, improper irrigation practices, such as over‐irrigation or poor application uniformity, can lead to water wastage and potential runoff, further intensifying water scarcity challenges in potato‐growing regions.

#### 3.3.2 Impact on SDG 6 (Clean Water and Sanitation)

The scenario analysis also reveals the positive impact on SDG 6 indicators (Figure 5C). As the consumption of potato and milk decreases, water consumption (aquatic ecosystems; ≈75.04%), FE (≈73.67%), freshwater eutrophication (≈75.98%), and GW (freshwater ecosystems; ≈74.97%) all show significant reductions. The results indicate that potato and milk production have significant impacts on freshwater ecosystems, particularly in terms of aquatic ecosystems and FE. The high values in these categories are primarily due to the increased water usage and chemical runoff associated with the production processes. This increased water usage can lead to reduced water availability for other uses, such as human consumption and sanitation, which is a critical concern for SDG 6. Scenarios 3 and 4, which represent lower levels of production of potato and milk, show a continued decrease in the impact values. This is a positive trend, as it indicates that potato and milk production can be managed in a more sustainable manner to support the goals of SDG 6. According to the literature, the current potato peel waste (PPW) management method of landfilling has a negative environmental impact by emitting greenhouse gases and poisonous leachates (Khanal et al. 2023). Additionally, even though industry employs a variety of waste management strategies, farmers sometimes indiscriminately dump cow waste into rivers and lakes. This release of slurry can lead to water pollution, affecting both human and animal drinking water sources. Furthermore, the decomposition of organic matter in the slurry can release gases such as methane and hydrogen sulfide, contributing to air pollution. Milk pollution in rivers can lead to the growth of harmful bacteria like *E. coli* , which kills marine organisms and is also harmful to human health (Girma et al. 2014). Excessive nutrients like nitrogen and phosphorus in the slurry can cause toxic algae blooms, which are deadly to certain marine animals and can also poison humans through contaminated shellfish (Ngatia et al. 2019).

#### 3.3.3 Impact on SDG 13 (Climate Action)

The analysis of the impact on SDG 13 (Figure 5D) indicators demonstrates that a shift toward increased millet and sorghum consumption can contribute to climate action and the protection of marine ecosystems. Fossil resource scarcity (≈74.92%), stratospheric ozone depletion (≈75.16%), and mineral resource scarcity (≈74.48%) are reduced by 75% in Scenario 4 compared to the baseline. The production of agricultural inputs like fertilizers and pesticides used in potato cultivation requires significant amounts of fossil fuels and mineral resources. Additionally, the transportation of potato and dairy products relies heavily on fossil fuels, further contributing to fossil resource scarcity. Overuse and inefficient recycling of these minerals can lead to mineral resource scarcity. For example, phosphate rock, a key mineral resource for fertilizers, is a finite resource with limited global reserves (Mata et al. 2023). Certain refrigerants and coolants used in milk production facilities can have significant greenhouse gas emissions that deplete the stratospheric ozone layer if not properly managed (Veiga et al. 2022; Vidican et al. 2023). OD allows more harmful UV radiation to reach the Earth's surface, with negative consequences for human health and ecosystems. Studies show that OD affects stratospheric ozone levels, leading to elevated UV radiation exposure, which can cause skin cancers and eye cataracts (Barnes et al. 2022; Kittipornkul et al. 2023).

#### 3.3.4 Impact on SDG 14 (Life Below Water)

Similarly, marine eutrophication (≈76.79%) and marine ecotoxicity (≈67.01%; Figure 5E) show reductions of approximately 67%–77% in Scenario 4. When agricultural nitrates leach into fresh and marine waters, it can lead to ETP in these habitats as well (Le Moal et al. 2019). AD is the process in which molecules such as sulfur dioxide (emitted from equipment combustion), nitrogen oxides (released from fertilizer spreading), and ammonia (generated from manure storage and spreading) undergo oxidation or hydrolysis in the atmosphere. This results in the formation of nitric and sulfuric acids, which then precipitate as acid rain, snow, or fog. A similar occurrence can transpire in soils, leading to the infiltration of substances into water (Huguet et al. 2023).

#### 3.3.5 Impact on SDG 15 (Life on Land)

Regarding the impact on SDG 15 (Figure 5B) the scenario analysis indicates that increased consumption of millets and sorghum can lead to significant reductions in land use (≈74.97%), ozone formation (terrestrial ecosystems; ≈74.98%), terrestrial acidification (≈74.98%), GW (terrestrial ecosystems; ≈74.99%), TE (≈78.84%), and water consumption (terrestrial ecosystems; ≈75%). Milk has the highest cooling energy requirements compared to other key materials, including meat. Additionally, vegetables, particularly potatoes, also require a significant amount of cooling (James and James 2010). This dominant use of refrigerant releases a group of chemicals known as halogenated hydrocarbons, e.g., chlorofluorocarbons (CFCs) and hydrochlorofluorocarbons (HCFCs) into the atmosphere. Scientific evidence clearly shows that emissions of CFCs have been damaging the ozone layer and contributing significantly to global warming. Raw potato production represents a significant impact share of feedstock production. This share ranges from 25% in depletion of fossil fuels to up to 94% in acidification. The acidification and terrestrial eutrophication from feedstock production are related to ammonia emissions from fertilizer application (Moretti et al. 2022). A study on potato production in India also revealed that the fertilization phase had the highest negative impact on the environment (Kumar et al. 2021).

The study highlights the superior environmental sustainability of millets and sorghum compared to potatoes and milk, emphasizing their potential as a more viable and sustainable dietary choice. By evaluating the environmental impacts of consumption patterns, policymakers can make informed decisions to advance SDG 12 on responsible production and consumption while also contributing to SDGs 3, 6, 13, 14, and 15.

Although millet and sorghum are more environmentally friendly, they can nonetheless produce adverse environmental effects if not managed appropriately. Sorghum and millet are vulnerable to contamination by aflatoxin‐producing molds, which pose serious health risks, including liver cancer, immune suppression, and stunted growth in children (Awuchi et al. 2021). While these grains generally have lower contamination levels compared to other cereal‐based products, improper storage still creates large risks. To mitigate this risk, it is essential to ensure thorough drying of the crops before storage and to use airtight, moisture‐proof containers to prevent fungal growth. Good agricultural practices such as crop rotation and careful handling during harvest can further reduce contamination (Neme and Mohammed 2017). Additionally, introducing non‐toxigenic strains of Aspergillus flavus can help inhibit the growth of harmful fungi (Divakara et al. 2014). Education and training for farmers on proper drying, storage techniques, and the dangers of aflatoxins are crucial for prevention (Ayeni et al. 2021). Finally, chemical treatments like hydrated sodium calcium aluminosilicate and certain chemopreventive agents can help reduce aflatoxin levels in contaminated grains (James and Zikankuba 2018). Implementing these strategies is vital for safeguarding public health and ensuring the safety of sorghum and millet consumption.

The findings from our study closely align with global trends in sustainable food systems but also highlight important region‐specific nuances. Similar to many global studies (Matthews et al. 2023; Coming 2024), this research emphasizes the unsustainable impacts of conventional food systems, particularly from dairy, livestock, and water‐intensive crops, reflecting worldwide concerns about greenhouse gas emissions, land degradation, and biodiversity loss. It supports the global shift toward climate‐resilient crops like millets and sorghum for their lower environmental footprint and higher nutritional value, echoing recommendations from certain initiatives. The LCA and the focus on environmental hotspots and SDG alignment follow established global methodologies. However, the study makes a unique contribution by deeply localizing its analysis to India, considering per capita consumption patterns and traditional diets, aspects often overlooked by Western‐centric models. It brings attention to underrepresented crops like millet and sorghum and discusses the dual role of dairy as both nutritionally important and environmentally intensive, a particularly critical issue for India as the world's largest milk producer. The detailed scenario analysis showing potential reductions of up to 62%–79% in environmental impacts through dietary shifts toward millets and sorghum is notably proactive, exceeding the scope of many global studies. Moreover, the study explicitly connects its findings to multiple SDGs (notably SDG 3, 6, 13, 14, and 15), offering a strong, holistic perspective that ties food, environment, and health together.

## 4 Implications for Practitioners and Policy Makers

Practitioners in the agricultural sector, particularly those engaged in dairy and tuber production, must adopt integrated and sustainable farming practices to address environmental challenges and ensure long‐term viability. In dairy production, transitioning toward pasture‐based or rotational grazing systems can significantly reduce the environmental footprint by enhancing soil carbon sequestration, improving animal welfare, and reducing feed‐related emissions. Reducing reliance on synthetic fertilizers and chemical‐intensive feed crops through the use of organic inputs, legume‐based fodder, and locally sourced feed can further lower greenhouse gas emissions and nutrient runoff. Additionally, implementing manure management systems such as anaerobic digesters or composting can mitigate methane emissions while producing renewable energy or organic fertilizer, thus contributing to circular nutrient flows. Enhancing overall resource efficiency through precision feeding, water‐saving technologies, and energy‐efficient milking and cooling systems is also critical. For tuber cultivation, particularly potatoes, sustainable farming practices should focus on optimizing inputs and preserving soil and water resources. Efficient irrigation methods like drip or sprinkler systems, coupled with soil moisture monitoring and scheduling tools, can dramatically reduce water wastage and improve crop productivity. The adoption of rainwater harvesting, contour farming, and mulching not only supports water conservation but also enhances soil moisture retention and prevents erosion. Emphasizing soil health through organic amendments, crop rotation, intercropping with legumes, and minimal tillage improves nutrient cycling and long‐term productivity while reducing the need for synthetic inputs. Pest and disease management strategies should favor integrated pest management (IPM) approaches that reduce pesticide use through biological controls, resistant varieties, and crop monitoring. Beyond the farm gate, sustainable practices across the potato value chain are equally important. Using energy‐efficient machinery and low‐emission equipment during land preparation, planting, and harvesting minimizes fuel consumption. During processing, transitioning to renewable energy sources such as solar (Freeman et al. 2025) or biomass along with energy‐efficient technologies (e.g., heat recovery systems and smart cold storage) can reduce the environmental burden significantly. Improved logistics, such as route optimization, bulk transport systems, and eco‐friendly packaging materials, further reduce emissions associated with transportation and distribution. Additionally, waste valorization strategies—such as converting potato peelings and unsold produce into biogas, compost, or animal feed—close the loop and promote a circular economy within food systems.

The integration of advanced technologies such as precision agriculture, remote sensing, the Internet of Things (IoT), and blockchain is transforming sustainable agriculture by enabling data‐driven decision‐making and resource optimization across the value chain (Achuthan et al. 2025; Nath 2024). In parallel, community‐driven efforts, such as those observed in online zero waste movements, enhance public engagement and emotional investment in sustainability through socially mediated interactions (Achuthan and Khobragade 2025). Precision agriculture uses GPS‐enabled equipment, soil sensors, and variable rate technologies to apply water, fertilizers, and pesticides only where needed, thereby improving productivity while reducing waste, emissions, and environmental contamination (Tahir 2024; Vinod Chandra et al. 2024). Remote sensing through satellite imagery and drones provides real‐time insights into crop health, soil conditions, and pest outbreaks, allowing for timely interventions and sustainable land management (Aziz et al. 2025). IoT devices like soil moisture probes, weather stations, and automated irrigation systems further enhance resource efficiency by enabling real‐time monitoring and automation of farm operations. Blockchain adds transparency and traceability by securely recording every transaction and production step in a digital ledger, helping producers meet certification standards and build consumer trust (Montecchi et al. 2019). Together, these technologies reduce waste, increase yields, lower environmental impacts, and support data‐driven sustainability reporting, thereby contributing to resilient, efficient, and SDG‐aligned food systems.

Practitioners should explore cultivating alternative oilseed crops, particularly in regions facing or at risk of water scarcity. Effective management of resources such as water, energy, and soil nutrients is critical. Practitioners should focus on water conservation techniques, such as drip irrigation and rainwater harvesting, to address water scarcity issues. Additionally, the use of renewable energy sources for agricultural operations can further reduce the carbon footprint. Promoting the cultivation and consumption of climate‐resilient crops such as millets and sorghum, which have lower environmental impacts and higher nutritional value, can be a strategic move. These crops require fewer resources and are well‐suited to the agro‐climatic conditions in many parts of India. Practitioners also need to adopt sustainable practices throughout the supply chain, from production to distribution and retail. This includes reducing food loss and waste, utilizing eco‐friendly packaging, and optimizing transportation logistics to minimize emissions.

Policy makers need to develop and implement policies that support sustainable agricultural practices and encourage the adoption of environmentally friendly technologies. This includes providing subsidies and incentives for farmers adopting sustainable practices, investing in agricultural research and development, and promoting awareness and education about sustainable farming. Establishing robust regulatory frameworks to govern the use of inputs such as fertilizers, pesticides, and water resources is essential. These regulations should aim to minimize environmental harm while ensuring agricultural productivity. Policies should also promote the use of organic farming practices and the reduction of chemical inputs. Investing in infrastructure that supports sustainable agriculture, such as efficient irrigation systems, renewable energy installations, and storage facilities that reduce post‐harvest losses, is crucial. Improved infrastructure can enhance the efficiency and sustainability of agricultural operations. Policy makers should promote dietary shifts toward less resource‐intensive foods like millets and sorghum. Public health campaigns, nutritional education programs, and incentives for consumers to choose sustainable food options can help drive this change. Integrating these crops into public food programs such as midday meals and public distribution systems can also support this transition. Encouraging research and innovation in sustainable agriculture and food systems is vital. This includes funding for research on climate‐resilient crops, sustainable farming practices, and the environmental impacts of different food systems. Collaboration between research institutions, government agencies, and the private sector can foster innovation and the dissemination of best practices. Implementing systems for monitoring and evaluating the environmental impacts of agricultural practices and food consumption patterns is important. This can involve the use of environmental indicators and metrics to assess progress toward sustainability goals and inform policy adjustments. Engaging in international collaborations and partnerships can help share knowledge, technologies, and best practices for sustainable agriculture. Learning from global experiences and adapting successful strategies to the Indian context can enhance the effectiveness of sustainability initiatives. By addressing these implications, practitioners and policymakers can work together to create a more sustainable agricultural system that aligns with the SDGs. This collaborative effort is essential for ensuring food security, improving nutritional outcomes, and protecting the environment for future generations.

Even though many of these recommended sustainable practices are known to practitioners, they face barriers to adoption, including high upfront costs, limited access to technology, and inadequate training. Expensive systems like precision irrigation and renewable energy are often seen as risky investments without assured returns, especially for small farmers. Additionally, challenges such as weak regulatory support, limited infrastructure, and a focus on short‐term yields hinder uptake. Financial incentives, subsidies, training, and stronger government support are needed to make these practices more accessible and practical, helping drive a shift toward sustainable agriculture and food security (Chandran et al. 2025).

## 5 Limitations and Future Work

This study primarily employs a production‐centric approach, focusing on the manufacturing stage of food products. Although production does cause the highest impact on the environment, the other life cycle phases such as transportation, consumption, and waste management that also contribute to environmental impacts have not been included in this work. Variations in farming practices and technological advancements across different regions and time periods are not fully accounted for, which may affect the generalizability of the results.

Future studies can explore a more holistic view of the environmental impacts. Expanding the study to include different regions with diverse agricultural practices and dietary patterns could enhance the applicability and relevance of the findings globally. Exploring the leveraging of digital tools and technologies, such as remote sensing and blockchain, could improve data accuracy and provide real‐time insights into environmental impacts. Going beyond the selected basket of products and including a wider variety of food products in the analysis can help identify other significant contributors to environmental impacts. Investigating the impact of consumer behavior, including food waste and preferences, can provide insights into more effective strategies for promoting sustainable consumption. Finally, evaluating the effectiveness of different policy interventions and sustainability initiatives can provide practical recommendations for achieving SDG targets and promoting sustainable food systems. Future research should also integrate social and economic assessments to understand the broader implications of dietary shifts and sustainable agricultural practices on communities. By addressing these limitations and exploring these future directions, subsequent research can build on the findings of this study to advance our understanding of sustainable food systems and contribute to global sustainability goals.

## 6 Conclusions

The study's comprehensive life‐cycle assessment highlights the substantial environmental footprint of five major product groups and eighteen specific food items within India's BoP food category. Key findings reveal that milk and potatoes, in particular, exert significant environmental impacts across multiple categories. Milk production, which contributes 60%–75% of the environmental load, is a major driver of ecosystem harm, human health risks, and resource depletion. Similarly, potato cultivation poses notable risks, including substantial contributions to terrestrial acidification (7.94E‐5), global warming (5.4E‐9), and water consumption (4.32E‐5). Other food groups, such as meat‐based products, exhibit high values in impact categories like ionizing radiation (IR) and ozone formation. Crop‐based products contribute significantly to land use and ecotoxicity, requiring a concerted effort to promote water‐efficient farming practices, enforce sustainable resource management policies, and encourage the adoption of alternative crops and technologies to safeguard water resources in vulnerable regions, while cereal‐based products are major contributors to mineral resource scarcity and marine eutrophication.

The study also conducted a scenario analysis to explore the potential benefits of increasing the consumption of millets and sorghum. The analysis indicates that shifting to these climate‐resilient crops could reduce environmental impacts by approximately 62%–79% across SDG 3, SDG 6, SDG 13, SDG 14, and SDG 15. The most significant reductions are seen in terrestrial ecotoxicity (78.84%), marine eutrophication (76.79%), freshwater eutrophication (75.98%), stratospheric ozone depletion (75.16%) and water consumption in aquatic ecosystems (75.04%), ozone formation (human health; 66.08%), and ionizing radiation (62.40%). Millets and sorghum not only offer substantial environmental benefits but also provide superior nutritional value, making them a viable and sustainable alternative to more resource‐intensive foods.

These findings underscore the urgent need to promote sustainable agricultural practices and encourage dietary shifts toward less resource‐intensive foods. To mitigate the identified environmental impacts, sustainable agricultural practices should prioritize soil health, emission reductions, and water efficiency to lessen the effects on the environment. High‐demand crops can use less water when drip irrigation and rainwater harvesting are used, and cereals can use less fertilizer when precision farming and integrated soil management are used. Agroecological techniques like agroforestry and polycultures promote resilience and biodiversity in livestock, while rotational grazing and methane‐reducing feed additives help reduce emissions. When used in tandem, these strategies improve resource efficiency, lessen environmental damage, and support sustainable food systems. Additionally, policy support in fostering these changes, advocating for incentives for sustainable farming, investment in agricultural research and innovation, and public awareness campaigns to encourage the adoption of sustainable dietary practices is critical.

## Author Contributions

**S. U. Parvathy:** conceptualization (equal), data curation (equal), methodology (equal), software (equal), validation (equal), writing – original draft (equal). **Vysakh Kani Kolil:** data curation (equal), methodology (equal), visualization (equal), writing – original draft (equal). **Krishnashree Achuthan:** conceptualization (equal), project administration (equal), supervision (equal), validation (equal), writing – original draft (equal), writing – review and editing (equal).

## Conflicts of Interest

The authors declare no conflicts of interest.

## Data Availability

The data that support the findings of this study are available from the corresponding author upon reasonable request.
